# Sodium channels and mammalian sensory mechanotransduction

**DOI:** 10.1186/1744-8069-8-21

**Published:** 2012-03-26

**Authors:** Ramin Raouf, Francois Rugiero, Hannes Kiesewetter, Rachel Hatch, Edith Hummler, Mohammed A Nassar, Fan Wang, John N Wood

**Affiliations:** 1 Molecular Nociception Group, Wolfson Institute for Biomedical Research, University College London, London WC1E 6BT, UK; 2 Pfizer KCL Pain Lab, Wolfson Centre for Age-Related Diseases, King's College London, London, UK; 3 DMMBPS, Seoul National University, Seoul 151-742, Korea; 4 Biomedical Science University of Sheffield, Sheffield S10 2TN, UK; 5 Department of Pharmacology and Toxicology, University of Lausanne, Lausanne 1005, Switzerland; 6 Department of Cell Biology, Duke University Medical Center, Durham, North Carolina 27710, USA

**Keywords:** Mechanotransduction, Sodium channels, Pain, Nav1.7, Nav1.8, ENaCs

## Abstract

**Background:**

Members of the degenerin/epithelial (DEG/ENaC) sodium channel family are mechanosensors in *C elegans*, and Nav1.7 and Nav1.8 voltage-gated sodium channel knockout mice have major deficits in mechanosensation. β and γENaC sodium channel subunits are present with acid sensing ion channels (ASICs) in mammalian sensory neurons of the dorsal root ganglia (DRG). The extent to which epithelial or voltage-gated sodium channels are involved in transduction of mechanical stimuli is unclear.

**Results:**

Here we show that deleting β and γENaC sodium channels in sensory neurons does not result in mechanosensory behavioural deficits. We had shown previously that Nav1.7/Nav1.8 double knockout mice have major deficits in behavioural responses to noxious mechanical pressure. However, all classes of mechanically activated currents in DRG neurons are unaffected by deletion of the two sodium channels. In contrast, the ability of Nav1.7/Nav1.8 knockout DRG neurons to generate action potentials is compromised with 50% of the small diameter sensory neurons unable to respond to electrical stimulation in vitro.

**Conclusion:**

Behavioural deficits in Nav1.7/Nav1.8 knockout mice reflects a failure of action potential propagation in a mechanosensitive set of sensory neurons rather than a loss of primary transduction currents. DEG/ENaC sodium channels are not mechanosensors in mouse sensory neurons.

## Background

The identity of the noxious mechanotransduction channels in sensory neurons remains elusive but increasing evidence, particularly from knockout mice, indicates that transduction of noxious stimuli are carried out by more than one channel [1]. Many candidates have been proposed, however none has fulfilled the requirements of a *bona fide *noxious mechanotransducer that is expressed in mechanosensitive neurons, is activated by high threshold mechanical stimulation, and can be modulated by inflammatory mediators [2]. The list of potential mechanotransducers include TRP channels, potassium channels (see [1] for review), and the novel channel family Fam38a and b (Piezo1 and 2) transmembrane proteins [3]

Some invertebrate sodium channels of the ENaC/DEG superfamily are mechanosensors [4-6] but it is still unknown if any mammalian epithelial or voltage gated sodium channels participate in noxious mechanotransduction. Evidence from knockout mice suggests that ASICs are not involved in sensory transduction [7]. Epithelial Na^+ ^channels (ENaC) are voltage-independent, Na^+^-selective ion channels composed of α, β and γ subunits; the α subunit is necessary for channel function [8]. ENaC channels can be activated by membrane stretch and shear stress [9-11]. In mammals, the mechanosensitive hair cells of the cochlea express ENaCs, but genetic deletion of αENaC does not perturb mechanotransduction in these cells [12].

Studies in DRG neurons show the presence of β- and γ subunits in both rat and mouse but report contradictory data about the presence of the α channels in these neurons. However, functional ASIC channels with which ENaC subunits can heteromultimerise are broadly expressed in DRG neurons [13]. β and γENaCs were found in sensory nerve terminals associated with Merkel cells, Meissner and small lamellated corpuscles in the skin [14] whilst all three ENaC subunits were detected in rat trigeminal sensory nerves [15], in rat mechanosensitive nerve afferents innervating the muscle spindles where they contribute to mechanotransduction [16]. The extent to which these sodium channels participate in mammalian sensory mechanotransduction remains unclear.

Voltage-gated sodium channels underpin the propagation of action potentials. However, whether they also contribute to mechanotransduction is not known. A number of Nav1.7 mutations result in various pain phenotypes in humans [17]. Two mutant mouse strains with targeted ablation of Nav1.8, Nav1.7 or both, exhibit an unequivocal insensitivity to painful mechanical pressure while retaining the ability to sense low threshold mechanical stimuli [18]. Voltage gated sodium channels have been shown to be mechanosensitive. Nav 1.4 and Nav1.6 have been reported to respond to mechanical stimulation (stretch) by irreversible hyperpolarizing shift in voltage dependence [19-21]. The Nav1.5 subtype responds to mechanical stretch by changes in voltage dependence of the channel, but the changes are reversible [19]. We sought to investigate the possibility that Nav1.7 and Nav1.8 might also contribute to noxious mechanosensation.

Here we used knockout mice to determine the contribution of sodium channels to mechanotransduction. We show that deletion of β and γ ENaC subunits does not alter acute mechanical or thermal pain thresholds. We show that transduction of mechanical stimuli in isolated sensory neurons is unaffected by the absence of Nav1.8 and Nav1.7 channels and that the inability to fire action potentials is likely the basis of the behavioural deficits in mechanosensation observed in these mice.

## Results

### ENaC β- and γ subunit null mutant mice have normal acute noxious sensory thresholds

**Figure 1:**
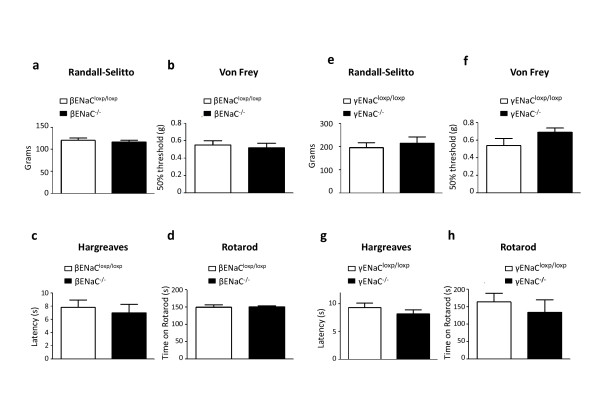
**Acute pain thresholds are normal in β and γ ENaC KO mice**. a) The Randall-Selitto test between βENaC^flox/flox ^and Nav1.8-Cre/βENaC^flox/**flox**^mice does not show any difference in behavior (t test, p = 0.6, n = 8 and 9 respectively). b, c and d) comparison of βENaC^flox/flox ^and Advillin-Cre/βENaC^flox/flox ^mice in the Von Frey test (t test, p = 0.64, n = 6 and 7 respectively), the Hargreaves test (t test, p = 0.6, n = 8 and 7 respectively) and the rotarod test (t test, p = 0.88, n = 4 and 4 respectively). e) Randall-Selitto test between γENaC^flox/flox ^and Nav1.8-Cre/γENaC^flox/flox ^mice (t test, p = 0.57, n = 10 and 10 respectively). f,g and h) comparison of γENaC^flox/flox ^and Advillin-Cre/γENaC^flox/flox ^mice in the Von Frey test (t test, p = 0.13, n = 11 and 13 respectively), the Hargreaves test (t test, p = 0.26, n = 13 and 12 respectively) and the rotarod test (t test, p = 0.56, n = 4 and 6 respectively).

β and γ ENaC subunits are expressed in all types of DRG neurons [14,15,22]. Noxious (high threshold) mechanosensation is sensed by small nociceptive Nav1.8-expressing DRG neurons [23]. To assess the possible role of β and γ ENaC in noxious mechanosensation, we therefore deleted them in Nav1.8-positive nociceptors using the Nav1.8-Cre mouse crossed to βENaC ^flox/flox ^and γENaC ^flox/flox ^mice. Nociceptor-specific ENaC-null mice did not exhibit any deficit in the Randall-Selitto test of mechanical pain (Figure 1a, e), suggesting that β- and γ ENaCs are not implicated in the transduction of painful mechanical stimuli.

The possible involvement of β and γ ENaC channels in low threshold mechanosensation (light touch) was studied using Advillin-Cre/βENaC ^flox/flox ^and Advillin-Cre/γENaC ^flox/flox ^mice, in which β or γ ENaC subunits are deleted in all DRG neurons. These mice allow us to assess the possible role of these channels in large DRG neurons responsible for low threshold mechanotransduction. Pan-DRG knockout of ENaC β- and γ subunits did not lead to any change in light touch sensation as measured in the Von Frey test (Figure 1b, f). These results combined with the Randall-Selitto data show that β- and γ subunits do not have a mechanotransducing function in DRG neurons.

Thermosensation was also tested in Advillin-Cre/βENaC ^flox/flox ^and Advillin-Cre/γENaC ^flox/flox ^mice using the Hargreaves test and was shown to be unaltered (Figure 1c, g). Motor coordination was also found to be unaffected by deletion of β- and γ subunits in DRG neurons (Figure 1d, h).

Altogether the results gathered from the behavioural assessment of mice in which β and γ ENaC channels were knocked out in DRG neurons reveal no obvious role for these channels in either acute pain or somatosensation.

Nav1.7/Nav1.8 double knockout (DKO) mice have elevated noxious mechanical thresholds but Nav1.7 and Nav1.8 are dispensable for mechanically activated currents in DRG neurons

We had previously reported the phenotype of the Nav1.8/Nav1.7 double knockout (*SCN10A*^cre/cre^:*SCN9A*^flox/flox^) mice which are refractory to noxious pressure [18]. The removal of Nav1.7 from nociceptors results in a small reduction in the TTX-sensitive and no change in TTX-resistant current densities. However, the input to the layer V wide dynamic range neurons in the spinal cord was substantially decreased upon noxious mechanical stimulation [24]. This raises the possibility that Nav1.7 and Nav1.8 are part of mechanotransduction protein complex, independent of their Nav channel function, and that they could be needed for the proper assembly or targeting of the noxious force transducer to the membrane or that they are transducers themselves. DRG neurons in culture respond to mechanical stimulation of the soma or neurites by eliciting inward mechanically activated (MA) currents [2,25,26]. In mouse DRG cultures, 50 to 60% of neurons are responsive to mechanical stimulation (depending on the recording configuration) under patch clamp recording conditions (data not shown but see reference [7]). Three types of MA currents are observed in response to focal stimulation; rapidly inactivating (RA) currents that are thought to correlate with low threshold mechanotransducers, intermediate and slow inactivating currents (IA and SA) that correlate with higher threshold mechanotransducers [2]. We have shown that NMB1, a conopeptide that specifically inhibits the SA type currents, increases the threshold responses to noxious pressure *in vivo *without affecting light touch [27].

**Figure 2:**
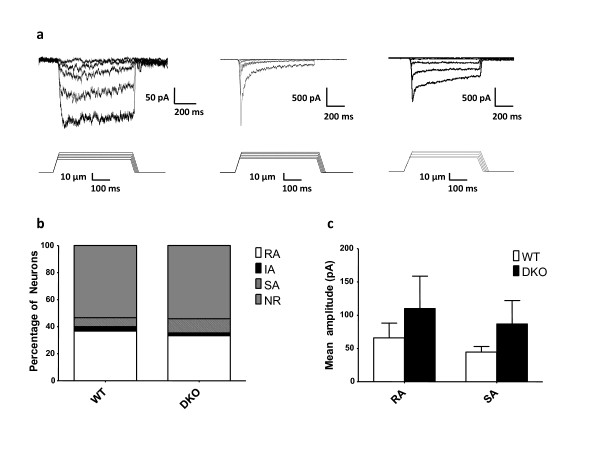
**Mechanically-activated currents are normal in Nav1.7/Nav1.8 DKO neurons**. a) Three types of mechanically activated currents are evoked in cultures of DKO DRG neurons Left to right, examples of SA, RA and IA currents are depicted. The mechanical stimulation protocol is shown below each trace. b) The bar graph depicts percentage of each type of current kinetics observed in DKO and wild type littermates. The percentage of each type of MA currents in DKO neurons was similar to that of the WT littermates (Chi square test, p = 0.9268). c) The mean amplitude of peak SA/IA and RA currents measured at 8 μm were not significantly different between the DKO and wild type neurons (one way ANOVA, p = 0.8167).

Therefore we compared evoked MA currents in Nav1.7/Nav1.8 double knockout (DKO) DRG neurons in culture to wild type (WT) responses. We recorded from a total of 30 WT and 48 DKO neurons and found that mechanical stimulation of the soma elicited MA currents in 49% (22 out of 48) DKO neurons compared to 47% (14 out of 30) for the WT neurons (Figure 2a, b). Within the mechanically responsive DKO population 33% (16/48), 2% (1/48), 11% (5/48) were of the RA, IA and SA types respectively (Figure 2b). The same proportions within recorded WT neurons were 37% (11/30), 3% (1/30) and 7% (2/30) respectively (Figure 2b). Hence DKO neurons have similar proportions of the three types of MA currents as the WT neurons.

We also assessed the contribution of the Nav1.7 and Nav1.8 to the amplitude of MA currents by comparing mean peak responses to mechanical stimulation in DKO and WT neurons. The overall amplitude of the MA currents tended to be larger, but not statistically significant, in DKO neurons compared to the WT. The peak RA and SA current amplitudes (measured at 8 μm displacement) were 110 +/- 49 pA and 87 +/- 35 in the DKO compared to 66 +/- 22 pA and 45 +/- 8.0 pA in WT neurons, respectively (Figure 2c). Overall we observed no deficits in MA currents in DKO DRG neurons, indicating that Nav1.7 and Nav1.8 are not mechanotransducer channels.

We did not distinguish between different types of DRG neurons (e.g. IB4 positive, capsaicin sensitive, etc). However, we assessed the action potential properties of the recorded neurons. It has been shown that narrow (duration less than 1 ms) action potentials are generally present in low threshold mechanoreceptors, while high threshold mechanoreceptors have wide, inflected action potentials [7,28,29]. In mouse DRG, 98% of wide action potentials are associated with TTX-r sodium currents that are mainly mediated by Nav1.8 TTX-resistant channels [7,28]. In our WT and DKO datasets, 10% (5/48) and 13% (4/30) of neurons had narrow action potentials respectively. In both cases we only observed RA type MA currents with 4/5 of WT and 2/4 DKO neurons responding to mechanical stimulation. Removing these neurons from the analyses did not alter the results significantly (data not shown).

### Nav1.8/Nav1.7 DKO neurons are deficient in their ability to generate action potentials

**Figure 3:**
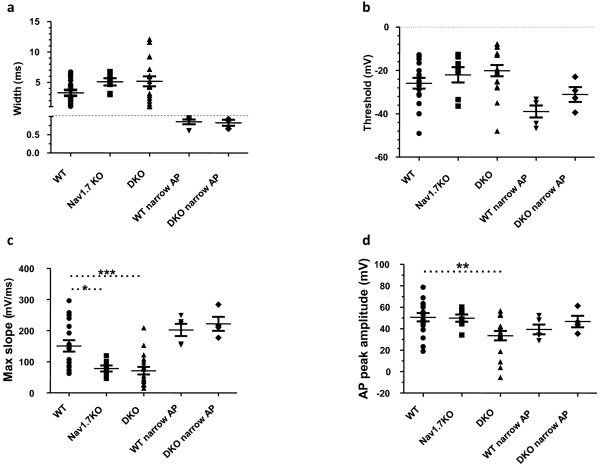
**Comparison of the properties of action potentials obtained in the three genotypes**. The properties of wide and narrow action potentials obtained from each genotype are shown. See materials and methods for calculation of the parameters. a, b, c, d) There was no significant difference between parameters of narrow action potentials in WT and DKO (ANOVA, p > 0.05, n = 4 and 5). c) The wide action potentials were slower in DKO and Nav1.7 KO compared to WT (One way ANOVA, p < 0.001, n = 18, 7 and 18 respectively). d) DKO action potentials had lower amplitudes than WT or Nav1.7KO (One way ANOVA, p = 0.02, n = 18, 17 and 7 respectively).

In order to understand the deficit underlying the pain transduction phenotype in DKO and Nav1.7 KO mice we compared the properties of the action potentials in each genotype. The properties of neurons that fired action potentials in each group are summarized in Figure 3. In total we included 18 WT, 18 DKO and 7 Nav1.7 KO neurons with wide action potentials. We analyzed the neurons with narrow action potentials (half-width less than 1 ms, 5 WT and 4 DKO) as a separate group since these neurons were not expected to be knockouts (Cre recombinase is only expressed in Nav1.8 expressing neurons.) The mean half-width and threshold of action potentials (APs) were similar in all groups (Figure 3a, b). However the maximum rate of rise (max slope) and the peak of the action potentials were significantly reduced in the DKO group (Figure 3c, d). The Nav1.7 KO neurons had action potentials that were slower to rise (max slope) but the peak of action potential was similar to the WT action potentials (Figure 3d). Hence the overall the tendency for the DKO neurons with wide action potentials (half-width greater than 1 ms) was to have slower and smaller action potentials compared to the WT.

While characterizing the properties of DRG neurons, we observed deficits in the ability of some DKO neurons to generate action potentials. Nav1.8 makes a significant contribution to the electrogenesis of action potentials in nociceptors while Nav1.7 is thought to be important in amplification of subthreshold potentials [30]. The contribution of Nav1.7 to the upstroke of action potentials in sensory neurons has not been reported.

**Figure 4:**
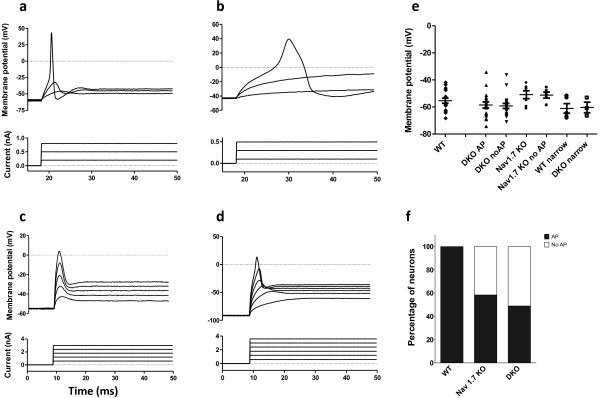
**Nav1.7/Nav1.8 DKO DRG neurons are deficient in their ability to generate action potentials**. a,b) Representative traces of action potentials (upper traces) evoked in response to depolarizing current injections (lower traces) are shown. Wide (a) and narrow (b) action potentials are observed in DKO neurons. c) Illustrates a representative example of graded responses to increasing depolarizing currents in a DKO neuron. d) Same cell as c, holding the neuron at -90 mV prior to depolarizing current injections resulted in generation of an all- or-none like action potential with a fast overshoot. e) Mean resting membrane potential for neurons with wide, narrow and no elicited action potentials (no AP) are shown. f) The bar graph shows the total percentage of neurons capable of firing action potentials in WT, Nav1.7KO and Nav1.7/Nav1.8 knock out mouse DRG neurons. Both DKO and Nav1.7KO groups are different from WT in having percentage of neurons not firing AP (Chi-square test p < 0.001 and p = 0.0072 respectively). Proportion of neurons firing action potentials in DKO or Nav1.7 KO are not significantly different from each other (Chi-square test p = 0.4306).

**Figure 5:**
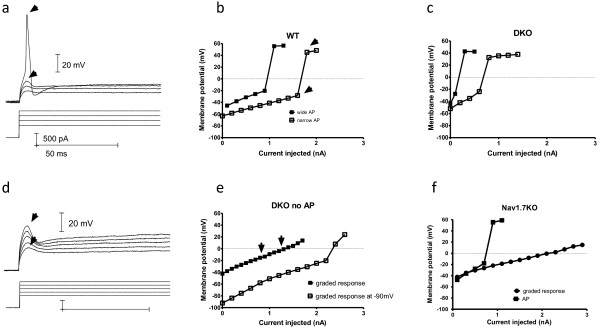
**Current-voltage relationship in cells that can generate all or none action potentials and those responding with small graded potentials**. a, b) Representative current-voltage (I-V) relationships in WT cell responding to increasing step current injections by eliciting all-or-none action potentials (a) is depicted in b (arrows). Generation of an action potential is marked by the non-linearity in the relationship at the point where additional stimulation does not result in significantly increased potentials (arrow). b, c) examples of I-V relationship for wide and narrow action potentials from WT and DKO neurons. d, e) I-V relationship for a DKO cell eliciting graded responses to step current injections depicted in e (arrows). Note the linear I-V relationship (e, closed squares) where no significant overshoot is present (d). Holding the same cell at -90 mV results in appearance of an overshoot with non-linear I-V relationship (d, open squares). f)In a Nav1.7 KO cell responding with small graded potentials the I-V relationship is linear hence all-or-none action potentials are not generated compared to a Nav1.7KO cell capable of generating action potentials.

In our experimental conditions injection of depolarizing currents from resting membrane potential elicited action potentials in 100% of WT neurons examined. Both narrow and wide action potentials with inflection were observed (not shown). We found that only 50% (24 out of 47) DKO neurons elicited narrow or wide action potentials (Figure 4a, b, and 4f). The DKO neurons that failed to elicit action potentials had a graded response to increasing depolarizing current steps (Figure 4c). It has been reported that 86% of Nav1.8 KO neurons fail to generate action potentials and instead elicit graded responses in a similar manner [30]. The differences between all-or-none action potentials and these graded responses in terms of the injected current to peak depolarization relationships are depicted in Figure 5. Action potentials have a clear non-linearity where the threshold of activation is crossed and an action potential is generated (Figure 5a, b). Further supra-threshold currents do not result in significantly larger upstroke peaks (Figure 5b, c). However, the graded potentials observed in a subset of DKO neurons had a linear current-voltage relationship with no clear all-or-none property since larger currents elicited larger voltage changes and no thresholds were detected (Figure 5d, e). We then tested the ability of Nav1.7 KO (*SCN10A *^cre/wt^:*SCN9A *^flox/flox^) neurons to generate action potentials and found that 4 out of 13 Nav1.7 KO DRG neurons failed to elicit action potentials in a similar manner (Figure 5f). The inability of a subset of neurons to generate action potentials when held at resting membrane potential could underlie the striking pain deficits seen in the Nav1.7 KO and DKO mice.

We had shown that there was an increase in TTX-sensitive sodium currents in Nav1.8 null DRG neurons [31]. To better understand the mechanism of failure to generate all-or-none action potentials in these DKO neurons we tested the responses to depolarizing current injections from a holding potential of -90 mV where the action potential amplitude has been shown to be maximal in Nav1.8 expressing neurons [32]. However, when the DKO neurons that failed to generate action potentials were held at -90 mV by injection of hyperpolarizing currents, they were able to generate action potential like responses with an overshoot (10 out of 10 neurons tested, Figure 4d). This change in behaviour is reflected in the current-voltage-relationship which becomes non-linear (Figure 5d, e). This suggests that in this subset of DKO neurons, removing inactivation of the remaining sodium channels by holding the neurons at hyperpolarized potentials could produce all-or-none like responses and hence, the main insufficiency in the DKO neurons is related to the lack of Nav1.8 and Nav1.7 sodium channels and not a general dysregulation of ion channels (e.g. potassium channels).

We further checked for a shift in the resting membrane potential in the DKO and Nav1.7 KO neurons as compared to the WT. We had complete datasets for 41 DKO, 12 Nav1.7 KO and 22 WT recordings which we included in this analysis. There were no significant differences between WT and either DKO or Nav1.7 KO resting membrane potentials but Nav1.7 KO neurons had a more depolarized resting potential than the DKO neurons (-55.3 +/- 1.9, -58.9 +/- 1.4 and -51.1 +/- 1.8 mV respectively, One way ANOVA, p = 0.016 with Bonferoni's multiple comparison test, n = 17, 38 and 12 respectively). There was no significant difference in resting membrane potential between WT and DKO neurons with narrow action potentials (61.1 +/- 3.2 and 60.4 +/- 3.9 respectively, t-test, p = 0.90, n = 5 and 4, Figure 4e).

Since holding DKO neurons at -90 mV changed graded responses to action potentials we looked to see if there were any differences in the resting membrane potentials amongst the cells. We divided the neurons in each genotype group based on their ability to fire action potentials (Figure 4e). We found no differences between resting membrane potential in neurons that did not fire action potentials and those that did (wide AP) in either DKO or Nav1.7KO groups (-57.0 +/- 1.2 vs -60.2 +/- 1.8 mV and -54.2 +/-2.4 vs -50.9 +/- 2.9 mV respectively, Student t-test, p = 0.80 n = 18 and 20, p = 0.94 n = 7 and 5 respectively). Further, no differences were found between Nav1.7KO and DKO neurons with graded responses although a trend for Nav1.7KO neurons to be more depolarized was noticed (Figure 4e, One way ANOVA, p = 0.217, n = 20 and 5 respectively), and no difference in resting membrane potential between neurons with wide action potentials of the three genotypes (Oneway ANOVA, p = 0.8559, n = 12, 5, and 6 respectively). These data indicates that the inability to fire action potential is not due to a shift in the resting membrane potential. Altogether the deficit in generation of all-or-none action potentials in a subset of Nav1.7 KO neurons and the fact that no difference was observed in the resting membrane potential between these cells and those Nav1.7 KO neurons firing action potentials strongly suggest that Nav1.7 directly (or indirectly) is required for the generation of the upstroke of action potentials in a subset of DRG neurons.

## Discussion

Here we tested the involvement of both voltage-gated and epithelial sodium channels in the transduction of mechanical stimuli using knockout mice. We have shown that β- and γ ENaC channels are not involved in acute mechanical pain and somatic mechanosensation. ENaC channels belong to the same family of ion channels as degenerins (DEG), which are heavily implicated in mechanosensation in the nematode *C. elegans *[4,6]. Mammalian ENaCs are clearly mechanosensitive and participate in transduction of mechanical stimuli in certain cell types, including vascular smooth muscle cells [33]. ENaCs expressed in Xenopus oocytes are activated by application of laminar shear stress [10]. They are also thought to participate in mechanotransduction in vagal nerve terminals of the aortic branch and carotid sinus [34] and nerve afferents innervating muscle spindles [16] as in these structures amiloride abolishes firing of action potentials. A plausible explanation for the lack of effect of deletion of the β and γ ENaC on behavioural responses to mechanical stimuli might be the absence of the channel forming α- subunit, or the possibility that ENaCs are involved in the subtle regulation of the transduction signal which may not be detected in our behavioural tests. Another explanation might simply be that different ENaCs are redundant and the knockout of one subunit is not sufficient to suppress sensory mechanotransduction. Generation of double knockout mice would be informative in this regard.

Several published observations support the lack of ENaC participation in sensory mechanotransduction: mechanically-activated currents in DRG neurons are insensitive to the potent ENaC channel blocker amiloride [25,26,35]. Also expression levels of ENaC mRNAs in mice do not match the developmental profile of mechanosensory acquisition and are relatively low in adults [22].

We have also showed that deletion of β- or γENaC does not impair thermosensation, as heat sensing was unaffected in ENaC-null mice. It has been reported that ENaC currents can be enhanced by cold temperatures [36]. However, at least one type of cold-activated current in DRG neurons is insensitive to amiloride [37]. It is therefore unlikely that ENaC channels play a major role in sensory thermosensation.

The stretch sensitivity of some voltage gated sodium channels would suggest a role in mammalian mechanotransduction. We tested the hypothesis that Nav1.7 and Nav1.8, alone, or in a complex, can contribute to the transduction of noxious mechanical stimuli. However, we found that Nav1.7 and Nav1.8 DKO DRG neurons respond to mechanical stimulation in a manner similar to the WT neurons, indicating that both channels are dispensable for mechanically activated currents. Interestingly, the proportions of neurons expressing different MA current types observed here are slightly different than those reported in adult mice [8]. This is most likely due to strain differences, as mechanical thresholds have been shown to be strain dependent [38]. However the proportions of neurons expressing a particular MA current type were similar in WT and DKO littermates. We reported previously an increase in the TTX-sensitive voltage gated sodium currents in the Nav1.8 null DRG [31]. It is unlikely that such increase would have masked the contribution of the Nav1.7/Nav1.8 particularly given the behavioural phenotype of the DKO animals. In our survey of mechanically activated currents in DKO vs WT DRG neurons in culture we did not subclassify the neurons as there are no robust methods available for distinguishing the DKO neurons from other neurons. However, over 85% of peripherin+ sensory neurons express Nav1.8 and ablation of these neurons results in resistance to mechanical pain [23] suggesting that the noxious mechanotransducer complex is expressed primarily in these neurons. The expression of Nav1.8/1.7 in the nociceptor endings where noxious sensory stimuli are transduced to action potentials, could suggest a physical link between the transduction and transmission channels. Indeed a number of cytoskeletal and other proteins have been shown to bind to Nav1.8/1.7 [39,40]. However, as we found the absence of these sodium channels does not affect the cellular transduction of mechanical stimuli, which suggests that the two types of channels operate independently of each other (also see [7]).

The restricted presence of Nav1.8/Nav1.7 in peripheral neurons suggests that the key to the observed deficits in sensing noxious pressure by the DKO mice could perhaps lie in the failure to transmit of action potentials. Nav1.8 is a major contributor to action potentials in nociceptors whereas Nav1.7 is generally thought to function in amplifying sub threshold depolarizations to facilitate generation of action potentials [41]. We asked if the DKO neurons are able to generate action potentials in response to depolarizing currents. We found that 50% of neurons in the DKO mice are unable to generate action potentials at normal resting potentials, instead responding with graded depolarisations proportional to the size of the current injected. In the Nav1.8 null DRG it has been reported that 76% of neurons are unable to fire action potentials [30]. The disagreement between the proportions is likely due to the recording conditions as Renganathan et al recorded shortly after plating the DRG neurons [30], whereas we have recorded 18 to 24 hours post plating for optimal mechanical assay conditions [26]. Renganathan et al also reported that only 84% of the WT (Nav1.8 +/+) neurons fired all-or-none action potentials but we found 100% of WT neurons to be able to fire action potentials.

Interestingly, we found that 30% of neurons in the Nav1.7 KO DRG were not able to generate action potentials in response to depolarizing stimulation if held at resting membrane potential. As we did not observe major differences in the resting membrane potential amongst DKO, Nav1.7 KO and WT neurons, these data suggests a potentially important role for Nav1.7, perhaps through modulation of Nav1.8, in generation of action potentials in a subpopulation of Nav1.8 expressing sensory neurons.

Hence the mechanism of the mechanosensory behavioural deficits in DKO mice may narrow down to the inability of a subset of DKO neurons to fire action potentials. However, we also found that action potentials in DKO neurons were slower to rise and lower in amplitude than the WT (Figure 3). Changes in the waveform of presynaptic action potential affects neurotransmitter release in different synapses (e.g. [42]). The amplitude of action potential could also have a profound impact on transmitter release (e.g. [43]). It is not clear how the slowing of action potentials concomitant with a decrease in amplitude will affect transmission and transmitter release from the nociceptive neurons in the DKO but it is likely that a defect in transmitter release would contribute to increased pain thresholds and inability to develop inflammatory pain [24].

Unlike the DKO, the amplitudes of action potentials were not affected in the Nav1.7 KO but the action potentials were slower on average. This is not surprising given the contribution of Nav1.8 to nociceptive action potentials [28]. Whether Nav1.7 plays a differential role in the propagation of action potentials depending on neuronal context is unclear. Expression of a mutant Nav1.7 channel, which results in hyperexcitability in DRG neurons, causes hypoexcitability in sympathetic neurons [44]. Interestingly, co-expression of Nav1.8 with the mutant channel restores the excitability of sympathetic neurons suggesting an interaction between Nav1.7 and Nav1.8 in determining the excitability of neurons [44]. The Nav1.7/Nav1.8 co-expressing sensory neurons are clearly required for electrical signalling associated with noxious mechanosensation. The functional role of the sensory neuron subset that expresses Nav1.7 in the absence Nav1.8 is yet to be defined.

## Conclusion

Using knockout mice and electrophysiological assays we show that epithelial sodium channel β and γ ENaC subunits, as well as voltage gated sodium channels Na_v_1.7 and Na_v_1.8 are not involved in transduction of noxious mechanical stimuli in sensory neurons. However, noxious mechanosensation does require action potentials generated by Nav1.7 and Nav1.8 in sensory neurons.

## Methods

### Generation of ENaC knockout mice

**Figure 6:**
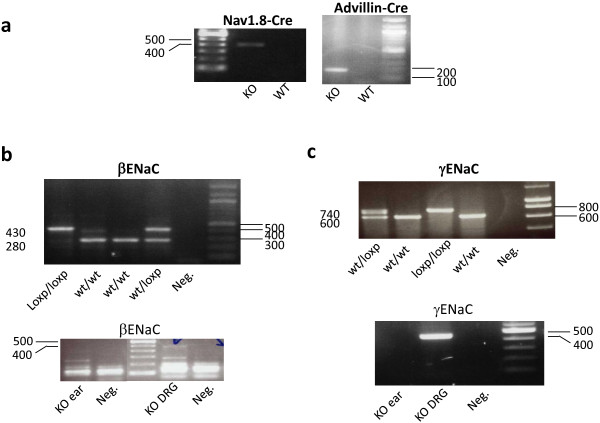
**Cre-mediated deletion of β and γ ENaC subunits in DRG neurons in mice**. a) PCR detection of the Cre recombinase band in genomic DNA from heterozygous Nav1.8-Cre (left) and Advillin-Cre (right) mice. b) Detection of floxed and WT bands in WT, heterozygous and homozygous βENaC^flox/flox ^mice (top) and of the KO band in the DRG but not the ear tissue of Nav1.8-Cre mice crossed to homozygous βENaC^flox/flox ^(bottom). c) detection of floxed and WT bands in WT, heterozygous and homozygous γENaC^flox/flox ^mice (top) and of the KO band in the DRG but not the ear tissue of Advillin-Cre mice crossed to homozygous γENaC^flox/flox ^(bottom).

The generation and genotyping of mutant mice with floxed alleles of βENaC (*SCNN1B*^flox/flox^) or γENaC (*SCNN1G*^flox/flox^) and Nav1.8-Cre or Advillin-Cre mice has been described previously [45-47]. Cre-mediated recombination in DRG cells was assessed using PCR of genomic DNA. The primers used to detect the floxed alleles were Scnn1b-s: 5'-CACTCAGGCACATGATAGACAGG-3' and Scnn1b-as: 5'-CTGCTCTGGGATTACAGG-3' for *SCNN1B *^flox/flox ^and Scnn1g-s: 5'-GCCTGATAAGAGAAGTCTG-3' and Scnn1g-as: 5'-TTGATGGAGACAGAGACGG-3' for *SCNN1G*^flox/flox^. Cre-mediated recombination in DRG neurons was assessed using primers Scnn1b-s and Scnn1b-ko: 5'-GATAAGGTGGGAAGAGCTGG-3' for βENaC and Scnn1g-s and Scnn1g-ko: 5'-CATAGACACAGCCATTGAAC-3' for γENaC. The presence of the Cre recombinase gene in the Nav1.8 locus was detected using primers SNS-s: 5'- TGTAGATGGACTGCAGAGGATGGA -3' and SNS-as: 5'- TTACCCGGTGTGTGCTGTAGAAAG-3' whilst the presence of the Cre recombinase gene in the Advillin locus was assessed using primers Avil-s: 5'- CCCTGTTCACTGTGAGTAGG-3' and Avil-as: 5'- AGTATCTGGTAGGTGCTTCCAG-3'. Nav1.8-Cre and Advillin-Cre mice were compared with βENaC ^flox/flox ^and γENaC ^flox/flox ^littermate controls. Mice knockout for βENaC and γENaC in small nociceptive DRG neurons were generated by crossing homozygous floxed exon 2 βENaC and γENaC mice [46] to heterozygous Nav1.8-Cre mice [24] whilst pan DRG βENaC and γENaC were generated by crossing homozygous floxed exon 2 βENaC and γENaC mice to heterozygous Advillin-Cre mice [45]. Mice were genotyped by PCR of genomic DNA. Nav1.8-Cre and Advillin-Cre heterozygous mice were positive for the presence Cre (band at 420 bp and at 180 bp respectively; Figure 6a). Wild type and floxed βENaC mice displayed bands at 280 bp and 430 bp respectively (Figure 6b, top) whilst wild type and floxed γENaC mice displayed bands at 600 bp and 740 bp respectively (Figure 6c, top) as expected from the primers used. Exon 2 was efficiently deleted in DRG of floxed ENaC/heterozygous Nav1.8 or Advillin-Cre mice as shown by the presence of the KO band (480 bp in floxed βENaC mice and 460 bp in γENaC mice in DRG but not ear tissue (Figure 6c).

### Generation of Nav1.8/Nav1.7 Double knockout (DKO) and Nav1.7 KO mice

The generation of the nociceptor-specific Nav1.7 knockout mice and Nav1.8/1.7 double knockout mice has been described previously [18,24]. Briefly, Nav1.7 KO (*SCN10A*^Cre/wt^:*SCN9A *^flox/flox ^) mice which are heterozygous for Nav1.8 Cre and homozygous for floxed Nav1.7 allele were crossed with each other to obtain *SCN10A*^Cre/Cre^:*SCN9A *^flox/flox ^(DKO) mice. The Nav1.8cre allele has a cre sequence inserted into the exon 1 of Nav1.8 followed by transcriptional stop signals hence mice that are homozygous for Nav1.8cre allele (*SCN10A*^Cre/Cre ^) are Nav1.8 knockouts [18]. Three genotypes of DKO, Nav1.7 KO and wild type (*SCN9a*^flox/flox^) at the expected ratios of 25%, 50% and 25% respectively were obtained. Genotyping was carried out by PCR as described previously.

### Behavioural tests

Behavioural tests were performed by experimenters blind to the genotype of the animals as described previously [47].

Randall-Selitto test of high threshold mechanosensation. Animals were placed in a restrainer and left to settle for a few minutes. Force was applied approximately midway along the tail. The force at which the animal attempted to withdraw the tail, vocalize, or struggle was recorded. The test was repeated three times for each animal. Results were expressed as the mean weight tolerated for each group.

Mechanical withdrawal threshold assessed using von Frey hairs. Static mechanical withdrawal thresholds were assessed by applying von Frey hairs to the plantar surface of the hindpaw. Unrestrained animals were acclimatized in acrylic cubicles (8 × 5 × 10 cm) atop a wire mesh grid for up to 60 min before testing. Calibrated von Frey hairs were applied to the plantar surface of the hindpaw until the fibre bent. The 50% withdrawal threshold was determined using the up-down method.

Hargreaves test of thermal nociception. Heat-pain threshold of the hindpaw was ascertained with the Hargreaves method using the Plantar Test. Unrestrained animals were acclimatized in acrylic cubicles (8 × 5 × 10 cm) atop a uniform glass surface for up to 60 min before testing. An infrared light source was directed onto the plantar surface of the hindpaw, and the latency to paw withdrawal was measured in seconds. Five responses were recorded for each animal on each testing occasion with at least 2 min between stimuli. To avoid tissue injury, the maximum stimulus latency was 15 s.

Rotarod test of motor coordination. Mice were placed on the rotarod as it was rotating at 5 rpm. After 30 sec, the rate of revolution was increased and reached a maximum of 40 rpm within 90 s. The duration that each animal spent on the rod was measured, with a cut-off time of 5 min. The test was performed three times for each animal with an interval of at least 15 min between each test.

### DRG Culture

DRG neurons were cultured as described previously [7]. The ganglia were dissected and treated with trypsin/collagenase enzyme mixture for 45 minutes. Mechanically triturated DRG mixtures were plated on laminin/poly-lysine coated culture plates. The culture medium contained D-MEM (Invitrogen), FBS, 10%; Glutamax (Invitrogen), and NGF (needed to maintain MA currents in cultures of adult DRG [2]).

### Electrophysiology

Recording of mechanically activated currents in DRG neurons has been described previously [7,26,48]. Experiments were carried out in whole cell configuration using axopatch 200B amplifier (Molecular Devices) controlled by pClamp 9 software. Recording pipettes were manufactured from borosilicate glass and had 1-2 MOhms resistance. In all recordings series resistance was compensated to 60-80% and periodically monitored. The cells were not used if series resistance was above 10 MOhms. Resting membrane potential was measured following membrane breakthrough in current clamp mode. Incremental mechanical stimulation of the soma was applied with a fire-polished glass micropipette attached to a piezo drive controlled by pClamp 9.0 software [6,8]. The probe was adjusted so that a 10 μm displacement did not make contact with the cell membrane but a 12 μm displacement caused a visible deformation of the membrane. The 10 μm displacement was taken as zero stimulation.

Pipette solution contained (in mM), KCl 140, HEPES 10, EGTA 10, Mg2+ 2, MgATP 2, Na2GTP 2. In some recordings KCl was replaced with KGluconate. External solution contained (in mM), NaCl 140, KCl 5, HEPES 10, Ca 1.0, Mg 2.0, pH to 7.4 with NaOH. All chemicals were purchased from SIGMA.

### Data analysis

Clampfit module of pClamp 9 software was used for the analysis of the electrophysiological data. Measurements of action potential duration and peak currents were carried out using the statistics module of Clampfit. The parameters measured were: half-width, referring to the width of action potential at 50% of the peak and the action potential amplitude, which was the voltage reached at the peak of the action potential. The threshold and maximum slope were measured from the first derivative of the voltage trace. The peak of the derivative within the upstroke was taken as the max-slope which was confirmed with the results from the statistics module in Clampfit. The threshold was taken from the voltage at the lowest minimum point of the derivative in the upswing phase which was confirmed from the measurement of the current step immediately prior to generation of the action potential. Statistical analyses of data were carried out using Graphpad Prism and Microsoft Excel software packages. Data are expressed as mean +/- standard error unless otherwise stated.

## Competing interests

The authors declare that they have no competing interests.

## Authors' contributions

RR carried out electrophysiological experiments with HK and co-wrote the manuscript. FR carried out behavioural phenotyping of ENaC mice with RH and co-wrote the manuscript. MAN generated the DKO mice. EH generated the floxed ENaC mice. FW generated the Adv:Cre mice. JNW conceived of the study, supervised the experiments and co-wrote the manuscript. All authors read and approved the final manuscript.
